# Localization of Cracks in Concrete Structures Using an Unmanned Aerial Vehicle

**DOI:** 10.3390/s22176711

**Published:** 2022-09-05

**Authors:** Hyun-Jung Woo, Dong-Min Seo, Min-Seok Kim, Min-San Park, Won-Hwa Hong, Seung-Chan Baek

**Affiliations:** 1 School of Architecture, Civil, Environmental and Energy Engineering, Kyungpook National University, Daegu 41566, Korea; 2 Department of Architecture, Kyungil University, Gyeongsan 38428, Korea

**Keywords:** unmanned aerial vehicles, crack, localization, concrete structure

## Abstract

Active research on crack detection technology for structures based on unmanned aerial vehicles (UAVs) has attracted considerable attention. Most of the existing research on localization of cracks using UAVs mounted the Global Positioning System (GPS)/Inertial Measurement Unit (IMU) on the UAVs to obtain location information. When such absolute position information is used, several studies confirmed that positioning errors of the UAVs were reflected and were in the order of a few meters. To address these limitations, in this study, without using the absolute position information, localization of cracks was defined using relative position between objects in UAV-captured images to significantly reduce the error level. Through aerial photography, a total of 97 images were acquired. Using the point cloud technique, image stitching, and homography matrix algorithm, 5 cracks and 3 reference objects were defined. Importantly, the comparative analysis of estimated relative position values and ground truth values through field measurement revealed that errors in the range 24–84 mm and 8–48 mm were obtained on the x- and y-directions, respectively. Also, RMSE errors of 37.95–91.24 mm were confirmed. In the future, the proposed methodology can be utilized for supplementing and improving the conventional methods for visual inspection of infrastructures and facilities.

## 1. Introduction

In recent years, there has been a rapid increase in the number of aging facilities in need of safety inspection, monitoring, and maintenance. Such inspection and maintenance involve huge social costs, indicating a pressing need for the development of economical and efficient technologies for the safety inspection of these facilities. Currently, safety monitoring and inspection of facilities in South Korea are conducted based on a visual inspection performed by trained and experienced inspectors. Among the different types of damages detected through visual inspection, cracks can provide important damage information as they are directly reflected in the structural health of the facilities. Therefore, the damage status of facilities is regularly monitored through visual inspection. However, the conventional method of human labor-intensive visual inspection is time-consuming and costly. In addition, as safety inspection is performed by human inputs, the detection of cracks is merely accurate. Moreover, subjective assessment is performed based on the experience and skills of the individual inspectors, resulting in the poor reliability and objectivity of the inspection.

To address the limitations of conventional visual inspection methods, active research on crack detection technology for facilities has been conducted based on an unmanned aerial vehicle (UAV) with efficient mobility and the capability of fitting various sensors. Long et al. [1] applied an extended cascading classifier (ECC) method to detect cracks on the surface of wind turbine blades based on UAV-captured images. Dongho et al. [2] used a deep convolutional neural network (DCNN) based on deep learning to detect cracks in facilities based on UAV-captured images. Shang et al. [3] developed an unmanned aerial system to inspect structures and used a deep learning-based convolutional neural network (CNN) to detect cracks in walls based on the images captured via the system. Salaan et al. [4] developed a UAV of a passive rotating spherical shell (PRSS) to detect cracks in structures based on UAV-captured images and used the next-generation robots for social infrastructure (NGRSI) systems. Muhammad et al. [5] utilized a speeded up robust feature (SURF) based feature detection algorithm to detect cracks in structures based on images taken by UAV-captured images. Ellenberg et al. [6] used both homography and crack identification algorithms to detect cracks in structures based on UAV-captured images. Chuncheng et al. [7] used a deep-learning-based crack detection on dam surface method to detect cracks in dams (CDDS) based on UAV-captured images. Li et al. [8] utilized the DenxiDeepCrack model to detect road cracks based on UAV-captured images. Bhowmick et al. [9] used computer vision and a deep learning based DCNN to detect cracks in the concrete surface based on UAV and still terrestrial video cameras. Several studies on UAV-based crack detection have been conducted, and the applicability of UAVs for crack detection has been confirmed through the aforementioned studies.

For the safety inspection of infrastructure and facilities, both the location and detection of cracks are crucial. In South Korea, information on cracks detected by human inspectors based on the design information of the facility is recorded in a visual inspection network diagram. The created visual inspection network diagram is utilized as data for facility repair and rehabilitation and a database for communication between stakeholders involved in a safety inspection. Therefore, the information on the crack location indicated in these diagrams is a critical factor. Nevertheless, when preparing the conventional visual inspection network diagram, as the information of crack location detected by inspectors is recorded based on a subjective judgment without significant details, the reliability and accuracy of the information are considered relatively low. Therefore, there is a need for studies on the localization of cracks using UAV-captured images and on crack detection. However, at present, compared with UAV-based crack detection studies, studies on the positioning of cracks are few.

Barber et al. [10] used a fixed-wing miniature air vehicle (MAV) to determine the GPS location of a ground-based object. The result showed that the target was located within 3 m of its actual GPS location. Minaeian et al. [11] used UAVs and unmanned ground vehicles (UGVs) and applied a customized motion detection algorithm to perform real-time target detection and localization. Trujillo et al. [12] used a simultaneous localization and mapping (SLAM) method for the position and orientation estimation of the target objects within a given range based on UAVs. The range of error obtained in the position estimation was a few meters. Hinas et al. [13] presented a target detection and localization algorithm using UAVs, and the target positioning error was estimated at the order of meters. Sun et al. [14] developed an all-in-one target detection and positioning system and used a fully autonomous fixed-wing UAV to perform experiments. The results showed that the error range of the target detection and positioning was approximately within the range of 0.8–13.9 m. Xun et al. [15] presented a UAV-based framework for tracking a mobile ground target, and the positioning error of the target was in the range of 8–28 m. Wang et al. [16] performed a study on estimating the position of the ground target using the revised Monte Carlo method based on the altitude of the UAV and the oblique angle of the lines of sight (LOS). Ma et al. [17] proposed a real-time object spatial localization method that combines binocular stereo vision and a global positioning system (GPS) based on insulator detection results. As described previously, several studies on the localization of target objects using UAVs have been performed. However, the range of the positioning error in most of these studies was in the order of a few meters. Moreover, the m-level positioning error cannot be utilized to locate cracks in real structures. Also, active research on vision technology applications in civil engineering fields is in progress. Studies such as detection, tracking, and localization of objects have been conducted using point cloud techniques, image stitching, and deep learning [18,19,20]. Moreover, possibility of using images for localization of cracks has been confirmed through these previous studies. However, few studies on the localization of cracks in concrete structures based on UAV-captured images have been reported. Therefore, this study aims to define the localization of cracks by using the relative position between objects in the image photographed by UAV. This study proposes a study on the localization of concrete cracks with mm-level position error by using the point cloud technique, image stitching, and homography matrix algorithm.

## 2. Materials and Methods

### 2.1. Overview

Most of the existing research on the localization of cracks using UAVs mounted the Global Positioning System (GPS)/Inertial Measurement Unit (IMU) on the UAVs to obtain location information. When such absolute position information is used, several studies confirmed that the positioning errors of the UAVs were reflected and were in the order of a few meters. To address these limitations, in this study, without using the absolute position information showing errors in the order of meters, the localization of cracks was defined using the relative position between objects in the UAV-captured images to significantly reduce the error level.

**Figure 1:**
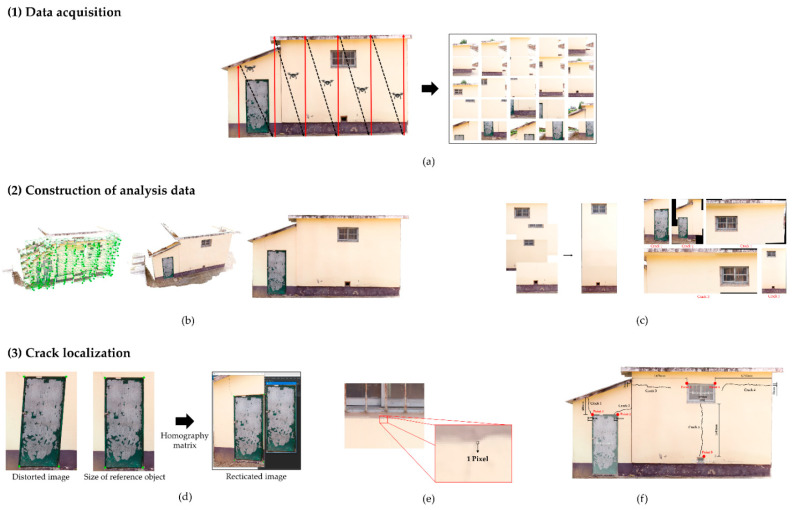
Overview of the UAV-based concrete crack localization: (**a**) Drone-based aerial photography; (**b**) Point cloud-based orthoimage generation for estimating sizes of reference objects; (**c**) Construction of image stitching-based reference object and crack visualization image data; (**d**) Image rectification using reference object size-based homography matrix; (**e**) Estimation of unit pixel size by defining the relationship of reference objects in orthoimage and crack images; (**f**) Estimating the relative position of cracks with respect to the reference object using the unit pixel size.

Figure 1 presents a schematic of the methodology for the UAV-based localization of cracks in concrete structures. The UAV-based concrete crack localization method consists of three steps as follows: (1) Data acquisition: drone-based aerial photography. (2) Construction of analysis data: (a) Point cloud-based orthoimage generation for estimating sizes of reference objects and (b) construction of image stitching-based reference object and crack visualization image data. (3) Crack localization: (a) Image rectification using reference object size-based homography matrix, (b) estimation of unit pixel size by defining the relationship of reference objects in orthoimage and crack images, and (c) estimating the relative position of cracks with respect to the reference object using the unit pixel size.

### 2.2. Drone-Based Data Acquisition

For the acquisition of image data through drone-based aerial photography, safety, flight control method, separation distance, and overlap must be considered.

In the photographing stage of concrete structure facilities, flight planning should be determined in advance to ensure safety during image acquisition using UAVs. Before the flight, the pilot should visually inspect the arrangement of the surrounding facilities and structures as well as the location of obstacles. Then, the UAV flight is controlled in the opposite direction to the obstacles. This is because the UAV can collide with adjacent obstacles during a flight. Therefore, it is safe to fly away from the obstacle.

The flight control modes of a UAV include autonomous, automatic, and manual flight. For autonomous flight, a method of measuring the relative distance to the sur-rounding environment using a camera and laser sensor termed SLAM is used. Automatic flight refers to the mode wherein a UAV flies automatically according to a predetermined fight path. This mode is primarily used to construct geospatial information in district units. This study uses photography at least 2-meters over distance from the target facility for the positioning of cracks.

In the acquisition of aerial images using UAVs to perform the localization of cracks in a facility, crack detection should first be conducted. If the separation distance between the UAV and facility is small, the image resolution increases, which is advantageous for detecting cracks. However, a small distance increases the risk of collision. Therefore, the separation distance and image resolution that ensure safety in the detection of cracks in a facility must be considered. To detect cracks in a bridge, Kim et al. [21] used a camera, which had a focal length of 15 mm and an image resolution of up to 20.8 MP, and employed a separation distance of 2 m. Similarly, to detect cracks in built infrastructures, Hoang et al. [22] used a camera, which had a focal length of 34.4 mm and an image resolution of up to 12 MP, and adopted a separation distance in the range of 1–2 m. To assess the crack of bridge fires, Liu et al. [23] used a camera installed in a UAV. It had a focal length of 15 mm and an image resolution of up to 20.8 MP, and the separation distance was set to 1 to 2 m. According to these studies, the performances of the cameras used to detect cracks in built infrastructures and facilities were achieved using a focal length, an image resolution, and a separation distance within the ranges of 15–34.4 mm, 12–20.8 MP, and 1–2 m, respectively. Therefore, in this study, the focal length of the camera used for the detection of cracks was 15 mm, the resolution of the acquired images was set to 20.8 MP, and the separation distance was set to 2 m.

To safely inspect facilities, the overlap setting for image acquisition should also be considered along with the safety of the image acquisition method using UAVs. Image overlap has a considerable implication for the construction of point cloud and image stitching analysis data [24]. Dandois et al. [25] compared the quality of a point cloud-based forest structure with different values of image overlap (%) and obtained high-resolution quality images with an overlap of 80% or more. Goodbody et al. [26] experimentally analyzed the applicability of aerial photogrammetry in forest areas according to image overlap using an image-based point cloud and obtained small data errors in an image overlap of 60% or more. To detect the lining surface anomalies of a tunnel using image stitching, Zhu et al. [27] set the overlap within the range of 30–50%. Similarly, to detect bridge cracks using image stitching, La et al. [28] set the overlap within the range of 30%. Moreover, to detect defects in structures using image stitching, Jahanshahi et al. [29] set the overlap to 50%. According to these observations, previous studies related to image overlap set the image overlap in the range of 30–80%. Considering this range, this study performed image acquisition with an image overlap set in the range of 50–60% for the construction of analysis data and the detection of damage information.

### 2.3. Construction of Analysis Data

In this study, the analysis data construction is performed in two steps: (a) Point cloud-based orthoimage generation for the estimation of the sizes of the reference objects and (b) reference object and crack visualization image data construction based on image stitching.

To estimate the size of a reference object using images obtained from drone-based aerial photography, orthoimage generation should initially be performed. Then, aerial images acquired through drone photography can be used to create a 3D model by applying a point cloud method. First, a geo-tagging process was performed wherein the information of a GPS/Inertial Navigation System location recorded in the flight log file was added as the attribute information of the aerial photography images. Then, corresponding points were extracted from the overlapping region of the image with the location information referenced. Next, point cloud data were created for the corresponding points extracted as a result of cross-referencing for the overlapping region. Finally, based on the created point cloud data, a mesh is formed and a 3D model is realized. A digital elevation model can be created based on the 3D model, and a 3D model with the same texture as the real model and orthoimage can be created by mapping the texture of the original photography image to that of the generated 3D model [30].

However, in a point cloud method, the image quality is lowered in the process of segmentation and reconstruction of images using points [31]. Thus, although the size of the reference object can be estimated in a 3D model created through this method, the detection of cracks with errors of the order of millimeters is challenging, limiting the delineation of cracks. Therefore, to achieve the quantitative localization of cracks, an image stitching method was employed in this study, and data that could combine the reference object and crack visualization images were constructed. A typical process of image stitching is performed in the following order: data input, feature point extraction between images, homography transformation, and image stitching [32]. In this study, a scale-invariant feature transform (SIFT) algorithm, which allows the extraction of feature points regardless of changes in the camera viewpoint, was used [33]. To combine two matched images, a homography matrix that defines the geometric relationship between both images is used. In this study, the homography matrix was calculated and applied using a RANdom SAmple Consensus (RANSAC) method to obtain an accurate model from the observation data.

### 2.4. Crack Localization

Crack localization is performed according to the following ways: (a) image rectification using a homography matrix based on the size of the reference object, (b) estimation of the unit pixel size by defining the relationship of the reference objects in orthoimage and crack images, and (c) estimation of the relative position of the cracks with respect to the reference object using the unit pixel size.

In the process of aerial photography using UAV, distortion may occur due to the parallax of the camera sensor.

**Figure 2:**
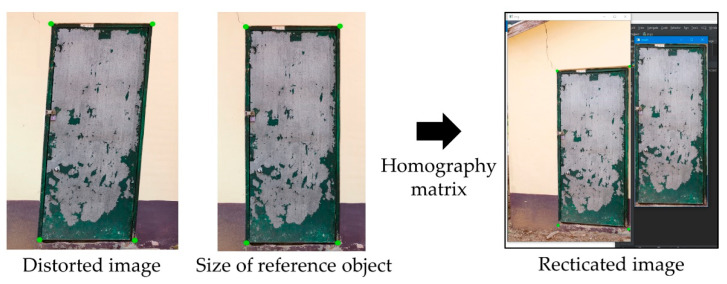
Image rectification using a homography matrix based on the size of the reference object.

In the process of image stitching before crack localization, if an image captured by a camera is used, image distortion may occur because of different parallax. By applying a homography matrix, it is possible to rectify image distortion due to parallax [34]. The feature points used in a homography matrix can be set with the size estimates of the reference objects using the point cloud-based orthoimage data as constructed in Section 2.3 [35]. Figure 2 shows image rectification using a homography matrix based on the size of the reference object.

**Figure 3:**
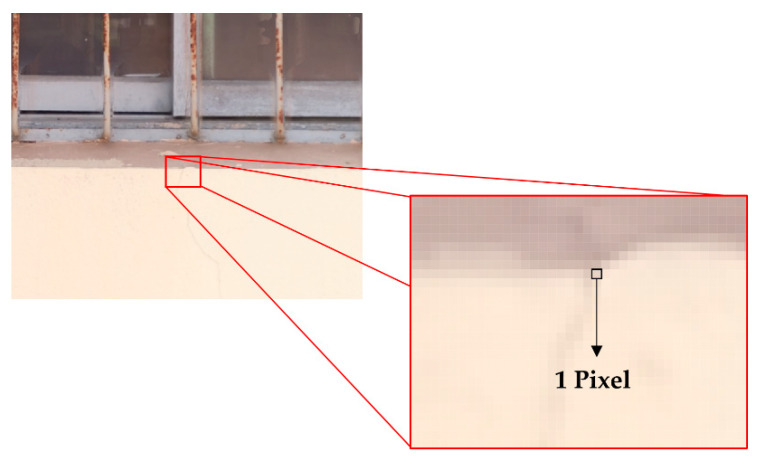
Unit pixel size for the analysis data image.

In the next step, based on the size of the reference object using the point cloud-based orthoimage data and the reference object and crack visualization image data, the unit pixel size was calculated. Figure 3 shows the unit pixel size of the analysis data constructed in this study.

Relative distance from a reference point of the reference object, defined using the unit pixel size of the crack calculated respectively from each of the rectified analysis data, is calculated using the (x, y) coordinates to estimate the relative position information of the crack. In addition, to verify the estimated values of the relative positions of the cracks, the values were compared with the location information of the cracks measured for a real facility using a measuring instrument.

## 3. Experimental Results

### 3.1. Data Acquisition

The UAV used in this study was DJI Inspire 2, and the camera model was Zenmuse X5S. The performance of the UAV used in this study for obstacle detection and collision avoidance was as follows: 5 m for the upward infrared sensor, 0.1–5 m for the downward vision system, and 0.7–30 m for the forward vision system. The field of View was up to 60° and 54° in the horizontal and vertical directions, respectively. The focal length of the camera lens was 15 mm, the image sensor dimension was 17.3 mm × 9.7 mm of CMOS 4/3”, and the resolution was 5280 × 2970 with a 16:9-aspect ratio, which allows image acquisition up to 20.8 MP.

**Figure 4:**
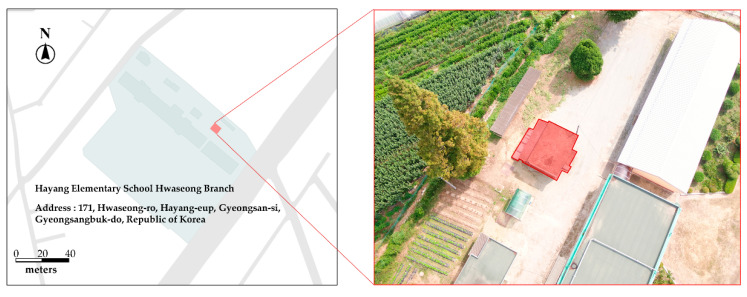
Overview of target site.

A study area was selected for the testing and verification of the proposed method of localizing cracks in a facility based on a rotary UAV of the Inspire 2 model fitted with a high-resolution camera. The target site was an aging annex building of an elementary school located in Gyeongsan-si, Gyeongsangbuk-do, Republic of Korea. Cracks and reference objects were identified on the exterior of the facility, enabling the estimation of the relative position of the cracks. The overview of the target site is shown in Figure 4.

To ensure safe flight and crack detection, considering the focal length of the camera and the acquired image resolution from previous studies discussed in Section 2.2, the separation distance was set to 2 m. In addition, image overlap was set in the range of 50–60% to acquire high-quality resolution. For the UAV-based image data acquisition, on 29 September 2019, a UAV flew across the exterior of the site following a grid pattern path. The flight mode was manual. Moreover, both the crack and the reference object were captured together in the same image. During the flight time of 7 min, a total of 97 images were acquired.

### 3.2. Construction of Analysis Data

**Figure 5:**
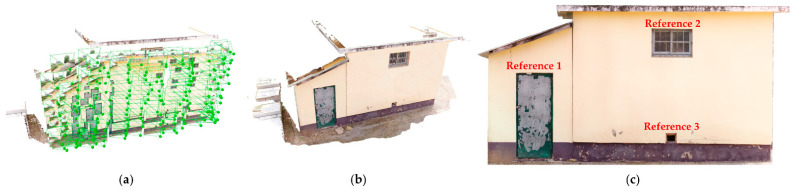
Process of orthoimage generation. (**a**) Initial processing; (**b**) Tie point matching and point clouding; (**c**) Orthoimage.

To estimate the size of the reference object, based on the acquired aerial photos, a point cloud-based orthoimage was created. Figure 5 shows the process of orthoimage generation, and the size of the reference objects through a 3D model analysis is presented in Table 1 below.

**Figure 6:**
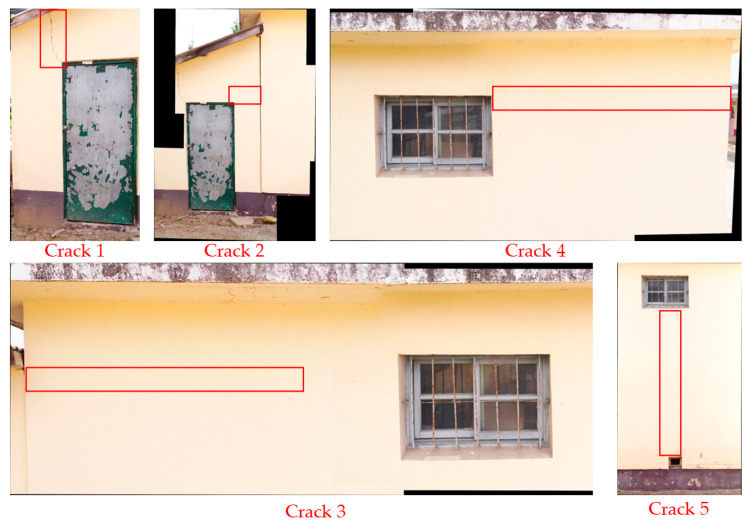
Analysis data including both reference object and cracks based on image stitching.

Next, data were constructed using both the reference object and the crack visualization image based on image stitching. Using the SIFT algorithm and the RANSAC method, data showing both the reference object and the crack were constructed. Five constructed images were obtained, and the analysis data showing both the reference object and cracks are illustrated in Figure 6.

### 3.3. Localization of Cracks in the Analysis Data

**Figure 7:**
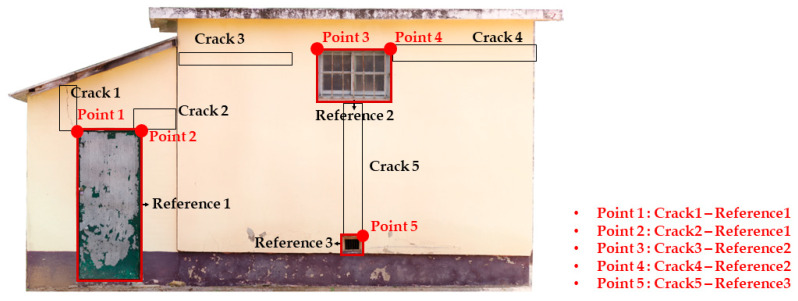
Analysis data after image rectification.

The above-mentioned constructed analysis data contained errors depending on the actual angle of the UAV photography. Therefore, in this study, the size of the reference object was estimated using a homography matrix, and the distorted analysis data were rectified. The analysis data after image rectification are shown in Figure 7.

In this study, the unit pixel size is required to estimate the relative position of the cracks. By using the analysis data after image rectification and the size of the reference object, the unit pixel size of the analysis data was estimated. The unit pixel size calculated for each analysis data is presented in Table 2.

By using the unit pixel size for each analyzed data in Table 2, the relative distance of the cracks from the reference point of the reference object was calculated. Then, to estimate the relative position of the crack, the starting and end points of the crack in the horizontal direction and those in the vertical direction were estimated from the reference point (0, 0) of the reference object. In this study, the starting and end points of the crack were expressed using the (x, y) coordinates derived in Section 2.4 to estimate the relative position of the crack. Table 3 outlines the relative positions of the cracks with respect to the reference object.

### 3.4. Data Validation

**Figure 8:**
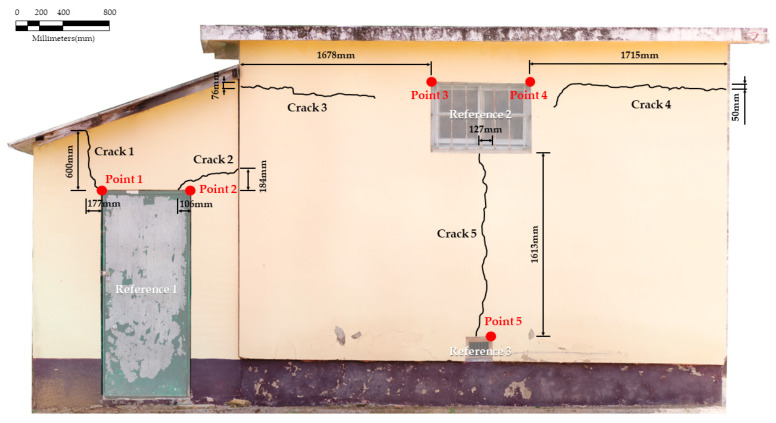
Visualization measured ground truth distance to cracks and reference object. (Location from reference point to remote points of cracks).

To test the value of the relative position of the crack estimated through the pixel size between the reference point and the crack, field measurements were conducted. Compared with the size of the crack, the measured ground truth was expressed in millimeters. A total of five measured reference points was obtained. The values of the ground truth are outlined in Table 4, and the visualization of the ground truth values is presented in Figure 8.

Next, the accuracy of the estimated relative position values was analyzed to verify the applicability of the proposed crack localization methodology. Accuracy analysis was performed by comparatively analyzing the above-mentioned estimated relative position values and the ground truth values obtained through field measurement. The results of the comparative analysis for the estimated relative positions of the cracks based on the proposed method in this study and the measured relative position values are presented in Table 5.

By comparing the estimated relative positions of the cracks with ground truth, errors of 24 to 84 mm on the x-direction and 8–48 mm on the y-direction were confirmed. Also, the RMSE errors of 37.95–91.24 mm were confirmed.

## 4. Discussion

In this study, to locate cracks in concrete objects using drone imagery, the relative positions of the cracks were experimentally estimated. UAVs can be the tasks of inspecting hard-to-reach locations and acquiring data. Due to these advantages, the application in facility safety inspections has recently attracted attention. However, like the study of Kim et al. [21], most of the current research focus on detecting the presence or absence of cracks. In addition, like the study of Trujillo et al. [12], the estimation of object location research using UAVs have limitations in application to facility safety inspection due to meter-level errors.

This study estimated the location of cracks with an error of 37.95–91.24 mm. Existing object localization research has been estimated based on GPS/IMU information of UAVs. In this study, without using the GPS/IMU information, localization of cracks was estimated using relative position objects based on drone imagery. In addition, mm-level position errors were obtained using point cloud technique, image stitching, and homography matrix algorithms. In this respect, this study is judged to be different from previous studies.

However, in some cases, sufficient or appropriate reference objects may not be available in the target concrete structure for the proposed research method to detect the location of cracks based on the reference object. In such cases, the calculation of the unit pixel size based on the reference object to estimate the relative position is challenging. Moreover, when the error level of the orthoimage is high, the measurement of the unit pixel size of the reference object may be inaccurate. Therefore, both the case of insufficient reference objects in the target structure and that of a high-error level in the orthoimage would be addressed in our future study. In addition, the methodology of this study will be applied to various structures as well as concrete structures, and a study on localization of damage using 3D reference objects will be conducted.

Many researchers have conducted research on detecting cracks in facilities using deep learning and UAVs [1,2,3,4,5,6,7,8,9]. In the future, the convergence of this study and crack detection technology is expected to make a significant contribution to safety inspection of facilities. Also, the level of the positioning error for cracks estimated through the proposed methodology is in the order of millimeters. This result is expected to have considerable applicability in crack detection using drones in the future. In this study, if the information of the reference object of the concrete structure for crack detection can be constructed, cracks can be detected and their location provided according to the needs of stakeholders.

## 5. Conclusions

In this study, based on drone imagery, cracks corresponding to the damage information of facilities were detected and their relative positions were estimated. The accuracy of the estimation was verified by comparing the estimated relative position values of the cracks and the measured ground truth values.

Using drone imagery, an orthoimage with a spatial resolution of a ground sample distance at 0.28 cm/pixel was constructed, and the size of the reference object was estimated. Data showing both the reference object and crack visualization image were constructed based on image stitching. Five cracks and three reference objects were defined. The relative positions of the cracks with respect to the reference object were estimated through unit pixel size estimation. By comparing the estimated relative positions of the cracks with ground truth, errors of 24 to 84 mm on the x-direction and 8-48 mm on the y-direction were confirmed.

In this study, the estimation of the relative position of the concrete cracks was per-formed using only drone imagery. In addition, crack detection and localization at the error level in the order of millimeters were possible using only drone images in situations where there is no information available on existing facilities. In the future, it is expected that the methodology proposed in this study can be utilized for supplementing and improving the conventional methods for visual inspection of infrastructures and facilities.

## Figures and Tables

**Table 1:** Size of the reference objects through analysis of 3D model.

Reference Objects	Dimension (Width × Length, Unit: mm)
Reference 1	792 × 1823
Reference 2	881 × 622
Reference 3	240 × 215

**Table 2:** Unit pixel size calculated for each analysis data.

Classification	Unit Pixel Size (Unit: mm)
Crack 1	0.58
Crack 2	0.62
Crack 3	0.61
Crack 4	0.61
Crack 5	0.57

**Table 3:** Location of cracks relative to reference object. (Location from reference point to remote points of cracks).

Classification	Relative Position (∆x, ∆y), (Unit: mm)
Crack 1-Reference 1 (Point 1)	(−145, 553)
Crack 2-Reference 1 (Point 2)	(−168, 202)
Crack 3-Reference 2 (Point 3)	(−1762, −84)
Crack 4-Reference 2 (Point 4)	(1796, −8)
Crack 5-Reference 3 (Point 5)	(1151, 1661)

**Table 4:** Values of measured ground truth distance to cracks and reference object. (Location from reference point to remote points of cracks).

Classification	Ground Truth Values (∆x, ∆y), (Unit: mm)
Crack 1-Reference 1 (Point 1)	(−177, 600)
Crack 2-Reference 1 (Point 2)	(−106, 184)
Crack 3-Reference 2 (Point 3)	(−1678, −76)
Crack 4-Reference 2 (Point 4)	(1715, −50)
Crack 5-Reference 3 (Point 5)	(−127, 1613)

**Table 5:** Comparison of estimated relative positions and measured relative positions values.

Classification	Relative Position (∆x, ∆y), (Unit: mm)				
	Coordinate	Ground Truth	Estimate	Error	RMSE
Crack 1-Reference 1 (Point 1)	x	−177	−145	−32	56.86
	y	600	553	47	
Crack 2-Reference 1 (Point 2)	x	−106	−168	62	64.56
	y	184	202	−18	
Crack 3-Reference 2 (Point 3)	x	−1678	−1762	84	84.38
	y	−76	−84	8	
Crack 4-Reference 2 (Point 4)	x	1715	1796	−81	91.24
	y	−50	−8	−42	
Crack 5-Reference 3 (Point 5)	x	−127	−151	24	37.95
	y	1613	1661	48	
